# Webinar Training: an acceptable, feasible and effective approach for multi-site medical record abstraction: the BOWII experience

**DOI:** 10.1186/1756-0500-4-430

**Published:** 2011-10-20

**Authors:** Chantal C Avila, Virginia P Quinn, Ann M Geiger, Tessa J Kerby, Meaghan St Charles, Kerri M Clough-Gorr

**Affiliations:** 1 Kaiser Permanente Southern California, Research & Evaluation, Pasadena, CA, USA; 2 Division of Public Health Sciences, Wake Forest University School of Medicine, Winston-Salem, NC, USA; 3 HealthPartners Research Foundation, Minneapolis, MN, USA; 4 Clinical and Outcomes Research, Lovelace Respiratory Research Institute, Albuquerque, NM, USA; 5 Section of Geriatrics, Boston University Medical Center, Boston, MA, USA; 6 Institute of Social and Preventive Medicine (ISPM), University of Bern, Bern, Switzerland; 7 National Institute of Cancer Epidemiology and Registration (NICER) Institute of Social and Preventive Medicine (ISPM), University of Zürich, Zürich, Switzerland

## Abstract

**Background:**

Abstractor training is a key element in creating valid and reliable data collection procedures. The choice between in-person vs. remote or simultaneous vs. sequential abstractor training has considerable consequences for time and resource utilization. We conducted a web-based (webinar) abstractor training session to standardize training across six individual Cancer Research Network (CRN) sites for a study of breast cancer treatment effects in older women (BOWII). The goals of this manuscript are to describe the training session, its participants and participants' evaluation of webinar technology for abstraction training.

**Findings:**

A webinar was held for all six sites with the primary purpose of simultaneously training staff and ensuring consistent abstraction across sites. The training session involved sequential review of over 600 data elements outlined in the coding manual in conjunction with the display of data entry fields in the study's electronic data collection system. Post-training evaluation was conducted via Survey Monkey^©^. Inter-rater reliability measures for abstractors within each site were conducted three months after the commencement of data collection.

Ten of the 16 people who participated in the training completed the online survey. Almost all (90%) of the 10 trainees had previous medical record abstraction experience and nearly two-thirds reported over 10 years of experience. Half of the respondents had previously participated in a webinar, among which three had participated in a webinar for training purposes. All rated the knowledge and information delivered through the webinar as useful and reported it adequately prepared them for data collection. Moreover, all participants would recommend this platform for multi-site abstraction training. Consistent with participant-reported training effectiveness, results of data collection inter-rater agreement within sites ranged from 89 to 98%, with a weighted average of 95% agreement across sites.

**Conclusions:**

Conducting training via web-based technology was an acceptable and effective approach to standardizing medical record review across multiple sites for this group of experienced abstractors. Given the substantial time and cost savings achieved with the webinar, coupled with participants' positive evaluation of the training session, researchers should consider this instructional method as part of training efforts to ensure high quality data collection in multi-site studies.

## Introduction

Medical record review is a common data collection method for conducting epidemiologic research. Although investigators in sites from various geographical locations often collaborate to include diverse populations to enhance generalizability of study results, idiosyncrasies of site data and differences in quality of data abstraction between sites can introduce variability. Abstractor training is a key element in minimizing interobserver variability to create reliable data collection procedures. The choice between in-person vs. remote or simultaneous vs. sequential abstractor training has considerable consequences for time and resource utilization. Advances in information technology have produced readily available, low cost, efficient alternatives to traditional training approaches. Faced with the challenges of collecting complex medical record data for a follow-up study of breast cancer treatment effects in older women (BOWII), we conducted a web-based (webinar) abstractor training session to standardize training across six individual Cancer Research Network (CRN) sites. Evaluation of this web-based platform for multi-site medical record review instruction would be valuable to researchers considering approaches to or planning abstractor training.

Web-based training is increasingly being used in educational and business settings as an effective, low-cost method to teach students and train employees. Although the literature suggests that online training is just as effective, or slightly more effective than in-person/classroom-based instruction for cognitive and procedural learning [1-3] participant-reported satisfaction levels of online instruction have been mixed [2,3]. For example, some studies have found participants reporting higher or comparable levels of satisfaction with online training courses when compared to in-person instruction [2,4] while other studies have found participants reporting less satisfaction with online instruction [1,5]. Differences in satisfaction with online instruction may be explained by participants' familiarity with the subject content and prior experience with this training modality [6,7]. Although some research has been conducted to assess the effectiveness of online medical and nurse training programs [8-11], very little literature has been published on the topic of medical record abstractor training [12,13] and to the best of the authors' knowledge no published studies have examined participant-reported effectiveness and satisfaction with web-based training for medical record abstraction. To address this lack in the literature, we report our experience with a simultaneous web-based medical record review training session for epidemiological research purposes across six study sites. The goals of this manuscript are to describe the web-based training session, its participants and participants' evaluation of webinar technology for abstraction training.

### Study Methods

The BOWII multi-site cohort study is a follow-up to the existing study cohort (BOWI) which extended data collection through five additional years of follow-up and added a comparison cohort. A detailed description of the BOWI sampling and data collection procedures has been published elsewhere [14]. BOWII included women 65+ years of age diagnosed between 1990 and 1994 with stage I or II breast cancer (N = 1405, breast cancer cases) and matched comparisons (N = 1405, women without breast cancer) followed for a maximum of 15 years. The comparison cohort was matched on breast cancer cases' age, study site, and breast cancer diagnosis year.

The study was conducted at six CRN sites in the United States of America (USA): Kaiser Permanente, Southern California; Group Health Cooperative, Seattle, Washington; Henry Ford Health System, Detroit, Michigan; HealthPartners, Minneapolis, Minnesota; Fallon Community Health Plan, Worcester, Massachusetts; Lovelace Health System, Albuquerque, New Mexico. The study protocol was approved by the institutional review board at each of the participating CRN sites. The CRN is a consortium of 14 integrated health care delivery systems with over 11 million enrollees. The overall goal of the CRN is to improve the effectiveness of preventive, curative, and supportive interventions for both major cancers and rare tumors.

### Medical Record Abstraction Instrument

BOWII data collection was conducted via medical record review and focused on capture of information related to follow-up care and late treatment effects. The study's electronic data collection system (DCS2) consisted of an ACCESS database requiring direct data entry of over 600 data elements covering five content areas: 1) demographics, 2) surveillance visits, 3) surveillance mammography, 4) recurrences and/or subsequent breast cancer diagnoses and 5) comorbidities. The DCS2 captured detailed information on breast cancer cases' follow-up visits and mammography screenings such as visit dates, reason for visits, type of practitioner seen during visits and whether a clinical breast exam was performed during visits. Also captured was whether women had a recurrence and/or second primary breast cancer as well as comorbidities existing before or developed after an initial breast cancer diagnosis. Comorbidities and invasive malignancies, including breast, were captured for matched comparison subjects. Instructions on how to identify and code each data element contained in the DCS2 was thoroughly documented in the DCS2 coding manual including the data element number, definition and synonymous terms, coding ranges and directives.

### Medical Record Abstractor Training

A single three-hour webinar session was held for all six data collection sites with the primary purpose of simultaneously training all study personnel and ensuring consistent approaches to abstraction across all sites. Webinar participants (hereafter referred to as participants) consisted of chart abstractors as well as other team members critical to the success of the study including study investigators and project coordinators. Participants either connected to the online training session from their own computers or from a computer set up in a conference room which projected the session onto a screen allowing simultaneous viewing by study participants.

Abstractor training was led by a single instructor who is a Registered Health Information Technician and Certified Tumor Registrar with over 10 years experience conducting research related medical record reviews including abstraction for the BOWI study. Abstractor training focused on medical record review and capture of data elements as defined in the coding manual and data-entered directly into the electronic DCS2.

Prior to the training session, participants from the sites were asked to pilot test several medical record abstractions using the DCS2. Both content and system issues identified during pilot testing were sent to the instructor prior to the webinar. These questions and resolutions were compiled into a standardized question and answer (Q&A) form and disseminated to participants for review in preparation for discussion and demonstration during the webinar.

The web-based training session involved sequential review of each data element outlined in the coding manual in conjunction with the display of the data entry field (including drop down menus and labels) in the DCS2. The instructor demonstrated how to navigate the data collection instrument and capture each of the data elements contained in the tool. The instructor then proceeded to address each question on the Q&A form, while simultaneously navigating through the DCS2 forms displayed on the screen. The instructor engaged trainees in discussion by providing helpful hints based on personal chart review experience and facilitated communication between participants by asking if they had any questions, comments and/or suggestions that they would like to share with the group. The goal of the discussion was to create a common understanding of the data elements to ensure consistency of data collection across abstractors and sites.

### Post-Webinar Training Evaluation

Post-training evaluation was conducted to assess the effectiveness of the webinar modality for data collection training and participant satisfaction with the training webinar. Following the conclusion of the webinar training, participants were contacted via email requesting their feedback on the training session and provided with the link to participate in the survey. Participant evaluations were completed anonymously online using Survey Monkey^© ^and took approximately 10 to 15 minutes per person to complete. (See Additional File 1 for participant survey.)

### Post-Webinar Training Support

Approximately one week after the webinar training, the instructor conducted a follow-up conference call with each of the six sites to resolve any remaining site-specific issues or new questions that arose with the commencement of data collection. Subsequently, the instructor was available via email to answer any questions. To share resolution of issues and ensure consistency of data capture across sites, the instructor held monthly multi-site conference calls. Prior to each conference call, the instructor compiled any new questions received from the sites, as well as resolutions to these issues, and distributed the updated Q&A form for discussion during the call. The Q&A form provided documentation of issues raised and decisions made, and was used as a source of reference material for the abstractors. Conference calls were held for the first six months of data collection, with the majority of questions being addressed during the first three months of data collection. These monthly calls were discontinued after six months as new issues became infrequent and abstractors became more experienced.

### Post-Webinar Technical Support

Each abstractor was issued his or her own copy of the DCS2 for data collection. Minimal technical support was required as almost all programming issues were identified and resolved during pilot testing. In the rare instance, when an abstractor did experience a technical difficulty, the problem was quickly resolved by the DCS2 developer at the lead site. Consequently, delays in data collection were minimal.

### Inter-Rater Reliability

Because the BOWII study included multiple abstractors at multiple sites, inter-rater reliability measures were conducted for each abstractor within each participating site. Inter-rater reliability was done approximately three months after the commencement of data collection and after completion of a minimum of 40 medical record reviews per abstractor (20 breast cancer cases and 20 comparisons). The inter-rater reliability electronic data capture system (IRR2) was developed in ACCESS with similar front end views as the DCS2 and contained a subset of 48 key data elements capturing reasons for end-of-study follow up, breast cancer recurrence, surveillance mammography, and comorbidity data.

A sample of ten (5 breast cancer cases and 5 comparisons) medical records completed by each abstractor were randomly selected and a designated independent rater from each site re-abstracted the 48 data elements into the IRR2 system to evaluate data quality. The database was uploaded to a secure website for download and analysis by the study statistician. The re-abstracted value for each data element was compared to the originally abstracted value and percent agreement was computed for each abstracted medical record and for the entire study.

## Results

Of the 16 people who participated in the webinar training, 10 (62.5%) completed the online survey. As reflected in Table 1, almost all (90.0%) of the 10 participants had previous medical record abstraction experience and nearly two-thirds reported over 10 years of medical record review experience. In addition, more than half (62.5%) of respondents with prior abstraction experience reported having experience abstracting directly into an electronic data collection system. Half of the respondents also reported having previously participated in a webinar, among which three had previously participated in a webinar for training purposes. Similarly, participants reported frequent use of the internet for common personal activities such as shopping/banking/downloading music/social media (Table 2).

**Table 1:** Participant Abstraction and Online Training Experience Prior to Webinar

Survey Question	Webinar Participants N = 10	
	**N**	**%**

**Abstracted medical records for research projects before**		
Yes	9	90.0
No	1	10.0
**Years of experience abstracting medical records for research**		
< 1 year	1	12.5
1 to 3 years	2	25.0
10 or more years	5	62.5
**Abstracted data from paper or electronic medical record directly into an electronic data collection system before**		
Yes	5	62.5
No	2	25.0
Not sure	1	12.5
**Previously participated in a webinar**		
Yes, for medical record abstracting for research	1	12.5
Yes, for another type of training	2	25.0
Yes, for non-training purposes	1	12.5
No	4	50.0

*NOTE: Counts may not sum to total due to missing data*.

**Table 2:** Webinar Participants Use of Internet (N = 10)

Internet Usage	Skype		Social Media		Download Music/ Video/Docs		Online Banking/ Shopping	
	**N**	**%**	**N**	**%**	**N**	**%**	**N**	**%**

**At least once a week**	1	12.5	5	62.5	3	42.9	6	75.0
**At least once a month**	0	00.0	1	12.5	2	28.6	1	12.5
**Couple times a year**	2	25.0	0	00.0	0	00.0	1	12.5
**Never**	5	62.5	2	25.0	2	28.6	0	00.0

*NOTE: Counts may not sum to total due to missing data*.

**Figure 1:**
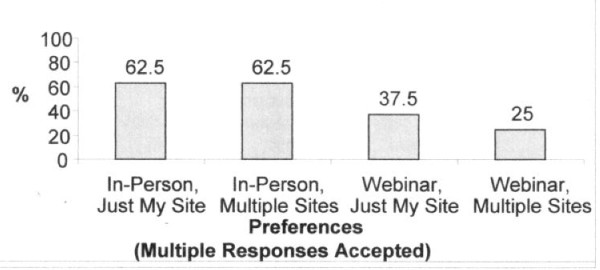
**Webinar Participants' Training Preferences**. Legend text: In-Person, Just My Site. In-Person, Multiple Sites. Webinar, Just My Site. Webinar, Multiple sites.

Table 3 reflects participants' evaluation of the webinar platform for abstraction training. All of the respondents rated the webinar as a good format for chart abstraction training. The majority of participants reported that the webinar training helped them to better understand the medical record abstraction content (87.5%) and use of the data collection system (75.0%). In addition, the majority of participants (87.5%) rated the webinar highly on its ability to facilitate discussion of questions and issues. All rated the knowledge and information delivered through the webinar as useful and reported that the webinar adequately prepared them for data collection. Almost three-quarters of participants reported the webinar training was more effective or just as effective for medical record review training relative to other types of training modalities. Nearly two-thirds of participants (62.5%) rated their ability to do a chart abstraction as "excellent" or "good" before the webinar versus 100% after the training webinar. Moreover, all participants reported they would recommend this platform for multi-site medical record review training. Consistent with participants' reports of the effectiveness of the webinar training, results of inter-rater agreement for data collection within sites ranged from 89.0 to 98.1%, with a weighted average of 95.0% agreement across sites (data not shown). Nevertheless, respondents reported a preference for in-person training over a web-based instructional approach, and site-specific webinar training over multi-site web-based training (Figure 1).

**Table 3:** Participant Rating of Webinar for Abstraction Training

Survey Question	Webinar Participants N = 10	
	**N**	**%**

**Webinar is a good format for chart abstraction training**		
Yes, very much	2	25.0
Yes, to some extent	6	75.0
**Webinar helped me better understand medical record abstraction content**		
Strongly agree	1	12.5
Agree	6	75.0
Neither agree nor disagree	1	12.5
**Webinar helped me better understand use of DCS2 data entry system**		
Strongly agree	2	25.00
Agree	4	50.00
Neither agree nor disagree	2	25.00
**Webinar format facilitated discussion of questions and issues**		
Strongly agree	3	37.50
Agree	4	50.00
Neither agree nor disagree	1	12.50
**Felt comfortable asking questions/participating in discussions during webinar**		
Strongly agree	4	50.00
Agree	3	37.50
Neither agree nor disagree	1	12.50
**Knowledge and information delivered through webinar**		
Very useful	4	50.0
Useful	4	50.0
**How well has the webinar prepared you for abstraction using the DCS2**		
More than adequate	6	75.00
Adequate	2	25.00
**Effectiveness of webinar format for abstraction training relative to other types of training**		
More effective	1	14.29
About the same	4	57.14
Less effective	2	28.57
**Ability to do a medical record abstraction using the DCS2 before webinar**		
Excellent	1	12.50
Good	4	50.00
Fair	3	37.50
**Ability to do a medical record abstraction using the DCS2 after webinar**		
Excellent	3	37.50
Good	5	62.50
**Would recommend webinar training for medical record abstractors working on future research projects that involve multiple sites**		
Yes	5	71.43
Probably	2	28.57

*NOTE: Counts may not sum to total due to missing data*.

## Discussion

Collecting complex medical record data presents considerable challenges in multi-site studies including standardization of data collection procedures, and time and resource utilization associated with in-person abstractor training. To address these issues, the BOW II study adopted webinar technology to orient and train abstractors from six diverse health plans across the USA. The webinar session proved to be an acceptable, feasible and effective component of a comprehensive effort to ensure high quality data collection.

Reports from the post-training survey suggest web-based instruction is a viable cost- saving alternative to in-person training with abstractors traveling to a central location or an instructor traveling to individual sites. For example, one-day in-person visits to the six participating sites could cost as much as six-thousand dollars compared to a cost of one-hundred and seventy-three dollars for a three-hour training webinar (assuming an average cost of $1,000 for airfare and one night's hotel accommodations for the instructor vs six cents a minute per caller for a three hour webinar). Webinar training also affords considerable time savings (e.g. minimum of 1 day per site for instructor travel vs three hours for a webinar).

Importantly, the results of the post-training inter-rater assessment support the effectiveness of using webinar technology as an integral part of training abstractors to produce reliable results. The webinar not only provided abstractors with consistent instruction and the ability to learn from others' questions, but it also fostered communication between participants at the various sites which set the stage for ongoing interactions between the abstractors. The rapport developed during the webinar facilitated open discussion during the subsequent multi-site Q&A calls, contributing to consistency of abstraction across sites and, in turn, resulting in high quality data collection. Of note, the supplemental support trainings would have been conducted as part of our comprehensive training approach regardless of the modality chosen to conduct the multi-site medical record review training.

In addition to the webinar being an effective training modality, it led to improvements in the coding manual and electronic data system (e.g. resolution of errors identified in the coding manual and inconsistencies between the coding manual and DCS2), as well as the identification of site-specific issues and the standardization of data collection procedures.

Our results are limited by the small sample size and may not generalize to abstractors with less experience in medical record review or lack of familiarity with the internet. There are distinct advantages to in-person training such as the opportunity to observe participants' performance and provide one-on-one instruction. It is also a more personal experience which may explain why participants stated a preference for in-person training. Nevertheless, given the high quality of data collection based on IRR results, coupled with the ability of the webinar to facilitate rapport between sites, the substantial time and cost savings achieved, and participants' positive evaluation of the webinar session, researchers should consider web-based training for use in multi-site studies.

## Conclusions

Conducting medical record abstraction training via web-based technology was an acceptable and effective approach to assist in standardizing a complex medical record review across six health plans. Researchers should consider this cost-effective instructional method as part of training efforts to ensure high quality data collection in multi-site studies.

## Competing interests

The authors declare that they have no competing interests.

## Authors' contributions

All authors have contributed to the development of the manuscript. AG and KCG co-designed the Post Webinar Training Evaluation Survey. TK and MSC conducted the literature review. CA conducted the data analysis, interpretation of results and the drafting of the manuscript. VQ, KCG and TK assisted in the interpretation of results. VQ and KCG partipated in substantive revisions to the draft manuscript. All authors have read and approved the manuscript.

## Supplementary Material

Additional file 1 **Post Webinar Training Evaluation Survey**. Description: Screen shots of post webinar training evaluation survey. Click here for file

## References

[B1] Rivera J Rice M A comparison of student outcomes & satisfaction between traditional & Web based course offerings Online Journal of Distance Learning Administration 2002

[B2] Schimming LM Measuring medical student preference: a comparison of classroom versus online instruction for teaching PubMed J Med Libr Assoc 2008 96 3 217 222 10.3163/1536-5050.96.3.007 18654658 PMC2479068

[B3] Goldberg HR Haase E Shoukas A Redefining classroom instruction Adv Physiol Educ 2006 30 3 124 127 10.1152/advan.00017.2006 16912147

[B4] Campbell M Floyd J Sheridan JB Assessment of student performance and attitudes for courses taught online versus onsite The Journal of Applied Business Research 2002 18 2 45 51

[B5] Carr Online Psychology Instruction is effective but not satisfying, study finds Chronicle of Higher Education 2002 46 27 A48, 2/5p

[B6] Chen YC Evaluating the learning effectiveness of using web-based instruction: An individual differences approach International Journal of Information and Communication Technology Education 2005 1 1 69 82

[B7] Carswell L Distant education via the Internet: the student experience British Journal of Education Technology 2000 31 1 29 46 10.1111/1467-8535.00133

[B8] Tuttle BD Von Isenburg M Schardt C PubMed instruction for medical students: searching for a better way Med Ref Serv Q 2009 28 3 199 210 10.1080/02763860903069839 20183016

[B9] Premkumar K Ross AG J L Technology-enhanced learning of community health in undergraduate medical education Can J Public Health 2010 101 2 165 170 20524384 10.1007/BF03404365 PMC6974159

[B10] Casebeer L Brown J Roepke N Evidence-based choices of physicians: a comparative analysis of physicians participating in Internet CME and non-participants BMC Med Educ 2010 10 10 42 10.1186/1472-6920-10-10 20537144 PMC2892500

[B11] Sisson SD Hill-Briggs F D L How to improve medical education website design BMC Med Educ 2010 21 10 30 10.1186/1472-6920-10-30 PMC2868857 20409344

[B12] Reisch LM Training, quality assurance, and assessment of medical record abstraction in a multisite study Am J Epidemiol 2003 157 546 551 10.1093/aje/kwg016 12631545

[B13] Liddy C Wiens M W H Methods to achieve high interrater reliability in data collection from primary care records Ann Fam Med 2011 19 1 57 62 10.1370/afm.1195 PMC3022047 21242562

[B14] Enger SM Breast cancer treatment of older women in integrated health care settings J Clin Oncol 2006 24 4377 4383 10.1200/JCO.2006.06.3065 16983106 PMC1913483

