# Supplementary material for: Webinar Training: an acceptable, feasible and effective approach for multi-site medical record abstraction: the BOWII experience
Source: BMC Res Notes. 2011 Oct 20;4:430. doi: 10.1186/1756-0500-4-430 (PMC3213762; doi:10.1186/1756-0500-4-430)
Supplement: Additional file 1 — Post Webinar Training Evaluation Survey. Description: Screen shots of post webinar training evaluation survey. [file 1756-0500-4-430-S1.PDF]

BOWII Abstractor Training Evaluation

1. Introduction and Assessment of Experience

Thank you for taking time to complete this survey. We believe the results will help others in the CRN and other settings develop useful and efficient approaches to abstractor training.

To protect your identity, results will be presented in aggregate form only. To increase your anonymity, we have not asked questions about your site and have included broad categories for any questions that ask about you.

Unless otherwise instructed, please pick the one best response to each question.

1. Have you abstracted medical records for research projects before?

- ◯ Yes
- ◯ No

BOWII Abstractor Training Evaluation

2. Questions for Abstractors with Prior Experience

Understanding your prior experience will help us interpret the results.

1. How many years of experience abstracting medical records for research do you have?

- ◯ <1 year
- ◯ 1 to 3 years
- ◯ 4 to 6 years
- ◯ 7 to 9 years
- ◯ 10 or more years

2. Have you ever abstracted data from a paper or electronic medical record directly into an electronic data collection system? Please answer yes if you were a BOW I or PROTECTS abstractor.

- ◯ Yes
- ◯ No
- ◯ Not sure

BOWII Abstractor Training Evaluation

3. Questions for Experienced Abstractors (cont)

1. How were you previously trained for other projects in which you abstracted medical records for research? Please check all that apply. Formal training would generally refer to training in which there was a written plan and specific material covered, whereas informal training would refer to on-the-job training where material is covered on an as-needed basis. In addition, formal training would tend to include group meetings whereas informal training might tend toward one-on-one discussions.

- ☐ Informal/on-the-job training with entirely local supervision
- ☐ Informal/on-the-job training with some supervision elsewhere
- ☐ Formal in-person training with local supervision
- ☐ Formal in-person training with some supervision elsewhere
- ☐ Webinar training in a multi-site project
- ☐ Limited or no training
- ☐ Other (please specify)

2. How would you rate the effectiveness of the webinar format for medical record abstraction training relative to other types of training you may have experienced?

- ○ More effective
- ○ About the same
- ○ Less effective

3. Would you recommend webinar training for medical record abstractors working on future research projects that involve multiple sites?

- ○ Yes
- ○ Probably
- ○ No

BOWII Abstractor Training Evaluation

4. General Webinar Questions

We want to start learning about your experience with the Webinar.

1. Have you previously participated in a webinar? Please check all that apply.

- ◯ Yes, for medical record abstracting for research
- ◯ Yes, for another type of training
- ◯ Yes, for non-training purposes
- ◯ No

2. Have you previously participated in video conferencing (not internet-based)? Please check all that apply.

- ◯ Yes, for medical record abstracting for research
- ◯ Yes, for another type of training
- ◯ Yes, for non-training purposes
- ◯ No

3. Have you previously participated in teleconferencing (not internet-based)? Please check all that apply.

- ◯ Yes, for medical record abstracting for research
- ◯ Yes, for another type of training
- ◯ Yes, for non-training purposes
- ◯ No

BOWII Abstractor Training Evaluation

5. Rating the Webinar's Impact on Preparedness for BOW II

1. Before the webinar, how would you have rated your ability to do a BOW II medical record abstraction using the DCS2?

- Excellent
- Good
- Fair
- Poor

2. After the webinar, how would you rate your ability to do a BOW II medical record abstraction using the DCS2?

- Excellent
- Good
- Fair
- Poor

BOWII Abstractor Training Evaluation

6. Rating Aspects of the Webinar

We would now like to ask about some specific parts of the webinar.

1. Please rate your agreement with the following statements:

	Strong agree	Agree	Neither agree nor disagree	Disagree	Strongly disagree
I could have done the BOW II medical record abstraction using the DCS2 without the webinar	◯	◯	◯	◯	◯
The webinar helped me better understand the BOW II medical record abstraction content	◯	◯	◯	◯	◯
The webinar helped me better understand the use of the DCS2 data entry system	◯	◯	◯	◯	◯
I feel I can now independently complete a BOW II medical record abstraction using the DCS2	◯	◯	◯	◯	◯

BOWII Abstractor Training Evaluation

7. Rating Aspects of the Webinar (cont)

1. Please rate your agreement with the following statements:

	Strong agree	Agree	Neither agree nor disagree	Disagree	Strongly disagree
The webinar format facilitated discussion of questions and issues	◯	◯	◯	◯	◯
I felt comfortable asking questions and participating in discussions during the webinar	◯	◯	◯	◯	◯
Simultaneous participation of all the sites enhanced my learning	◯	◯	◯	◯	◯
I will need additional support from local staff to complete BOW II medical record abstracts with the DCS2	◯	◯	◯	◯	◯
I will need additional support from staff from other sites to complete BOW II medical record abstracts with the DCS2	◯	◯	◯	◯	◯

BOWII Abstractor Training Evaluation

8. Rating Aspects of the Webinar (cont)

1. How would you describe the length of the webinar?

- ◯ Too short
- ◯ Just about right
- ◯ Too long

2. How would you rate the technical ease of participating in the August 4 BOW II abstractor training webinar?

- ◯ Very easy
- ◯ Fairly easy
- ◯ Neither easy nor hard
- ◯ Fairly hard
- ◯ Very hard

BOWII Abstractor Training Evaluation

9. Your View on Webinars as a Training Tool

1. Do you think a webinar is a good format for training to do medical record abstraction for research?

- ◯ Yes, very much
- ◯ Yes, to some extent
- ◯ No, not a very good format

2. Which of the following approaches to medical record abstraction training do you prefer? Please check all that apply.

- ☐ Webinar with just my site
- ☐ Webinar with multiple sites
- ☐ In-person training with just my site
- ☐ In-person training with multiple sites
- ☐ Other (please specify)

10. Overal Rating of the Webinar

This is the next-to-last set of questions, and the final set about the webinar.

1. Overall, how would you rate the knowledge and information delivered through this webinar?

- Very useful
- Useful
- Somewhat useful
- Not very useful

2. Overall, how would you rate how well the webinar prepared you for BOW II medical record abstraction using the DCS2?

- More than adequate
- Adequate
- Somewhat inadequate
- Very inadequate

3. Please share any recommendations you have to improve future webinar or other training for medical record abstraction for research.

5
6

BOWII Abstractor Training Evaluation

11. About You, the Final Questions

1. How often do you use the following:

	At least once a week	At least once a month	A couple of times a year	Never
Skype or other similar voice or video communication over the internet	◯	◯	◯	◯
Social media, such as Facebook, MySpace, Twitter, instant messaging, or video gaming	◯	◯	◯	◯
Dowloading music, video, documents, or other content	◯	◯	◯	◯
On-line shopping; banking; managing investments or insurance benefits; or similar	◯	◯	◯	◯
Word processing, spreadsheets, or other program at WORK	◯	◯	◯	◯
Word processing, spreadsheets, or other program NOT at work	◯	◯	◯	◯

2. How would you rate your computer skills compared to other abstractor colleagues?

◯ More skilled

◯ About the same

◯ Less skilled

3. How old are you?

◯ 29 or younger

◯ 30 to 49

◯ 50 or older

BOWII Abstractor Training Evaluation

12. A Last Chance to Provide Your Input

1. Is there anything else you would like to share with us?

5

6

BOWII Abstractor Training Evaluation

13. Conclusion

Thank you for taking time to share your impressions and thoughts with us. When a summary of the results is available, we will share it with the abstraction team.
